# Laparoscopic Approach to Primary Splenic Cyst: Case Report and Review of the Literature

**DOI:** 10.3390/life14010120

**Published:** 2024-01-14

**Authors:** Razvan Calin Tiutiuca, Alina Ioana Nastase Puscasu, Nicoleta Stoenescu, Mihaela Moscalu, Costel Bradea, Iuliana Eva, Cristian Dumitru Lupascu, Luminita Ivan, Madalina Maria Palaghia, Denisa Ioana Prisecariu, Eugen Târcoveanu, Andrei Vâță, Valentin Bejan, Alin Mihai Vasilescu

**Affiliations:** 1 Faculty of Medicine, University of Medicine and Farmacy “Gr. T. Popa” Iasi, 700115 Iasi, Romaniaandrei.vata@umfiasi.ro (A.V.); alin.vasilescu@umfiasi.ro (A.M.V.); 2 Surgery Department, Regional Institute of Oncology Iași, 700483 Iasi, Romania; 3 Faculty of Psychology and Education Sciences, “Alexandru Ioan Cuza” University of Iași, 700506 Iasi, Romania; 4 Radiology Department, “Dr Iacob Czihac” Military Emergency Hospital of Iași, 700483 Iasi, Romania; 5 Pathology Department, “Dr Iacob Czihac” Military Emergency Hospital of Iași, 700483 Iasi, Romania

**Keywords:** laparoscopy, splenic cysts, pseudocyst

## Abstract

Splenic cysts are rare benign lesions of the spleen, often asymptomatic and incidentally discovered during imaging studies. While many splenic cysts remain asymptomatic and do not require intervention, surgical management becomes essential in cases of symptomatic cysts, large cysts, or when malignancy cannot be ruled out. Laparoscopic surgery has emerged as a minimally invasive and effective approach for treating splenic cysts, offering advantages such as shorter hospital stays, reduced postoperative pain, and faster recovery. In this case report, we describe our experience with laparoscopic surgery for a symptomatic splenic cyst in a young patient.

## 1. Introduction

Splenic cysts are rare pathologies characterized by fluid-filled cavities within the spleen [1]. While they account for a small fraction of all splenic masses (0.07–7%), their diagnosis and management often pose a significant challenge in clinical practice given their rarity and nonspecific clinical presentation [1,2]. The rarity of splenic cysts often results in diagnostic challenges and necessitates a better understanding of their classification, aetiology, presentation, diagnostic modalities, and management. This article provides a detailed review of splenic cysts, their classification, aetiology, clinical presentation, diagnostic modalities, and management, with emphasis on recent advancements and emerging therapies.

## 2. Case Presentation

We report the case of a 38-year-old male patient, with no personal pathological history, who presented with a feeling of discomfort in the left hypochondrium and low intensity, intermittent pains, which appeared about 2 years ago, progressively worsening. He denies any previous abdominal or chest trauma.

During the objective examination, only a slight discomfort at palpation is observed in the left hypochondrium.

**Figure 1:**
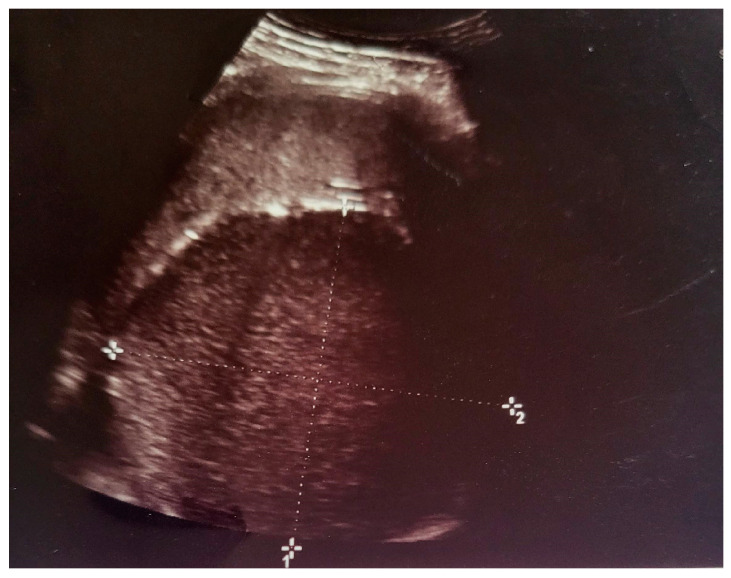
Ultrasound examination. The spleen with an area measuring 10/90 cm hypoechoic, homogeneous, well delimited, suggestive of an intrasplenic cystic lesion.

In this context, an abdominal ultrasound examination is performed that describes a hypoechoic, homogeneous, splenic area, measuring 10/90 cm, well delimited by a wall having a thickness of 0.2–0.4 cm, highly suggestive of an intrasplenic cystic lesion (Figure 1).

Subsequently, an MRI examination was performed that describes the liver within normal limits, presenting in VIIth a f 8 mm diameter lesion, T2 hypersignal, T1 hyposignal, without diffusion restriction, with centripetal nodular gadolinophilia in arterio-venous time, homogeneous hypersignal in the equilibrium phase, suggestive of capillary hemangioma.

The gallbladder and common bile duct are without lacunar images, and intrahepatic bile ducts are non-dilated. The portal vein is 12 mm in diameter. The pancreas is within normal limits, without native and postcontrast signal changes. The adrenal glands and kidneys bilaterally are without native and postcontrast signal changes.

**Figure 2:**
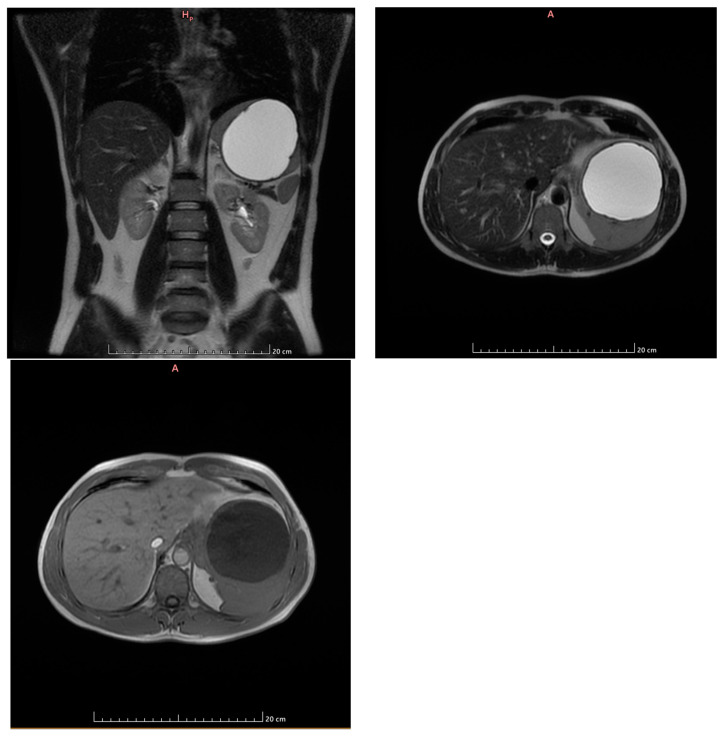
MRI examination (coronal T2, axialT2, axialT1 postcontrast). Voluminous fluid lesion of 10 cm diameter, with a discretely irregular thin wall, homogeneous fluid content, hypersignal T2, T1 hyposignal, without gadolinophilia.

The spleen is enlarged in volume, 137/113/136 mm (anteroposterior/transversal/craniocaudal), containing a voluminous fluid lesion of 10 cm in diameter, with discrete irregular thin wall, homogenous fluid content hypersignal T2, hyposignal T1, without diffusion restriction, without gadolinophilia (Figure 2).

The accessory spleen was also described, having 7 mm diameter, adjacent to the lower pole of the spleen, and there was an absence of bilateral lumboaortic and iliac adenopathies.

Conclusions of the MRI scan:

- Capillary hemangioma hepatic segment VII of 8 mm diameter.
- Grade I splenomegaly with simple voluminous splenic cyst, 10 cm in diameter.
- Accessory spleen adjacent to the lower pole of the spleen.

**Figure 3:**
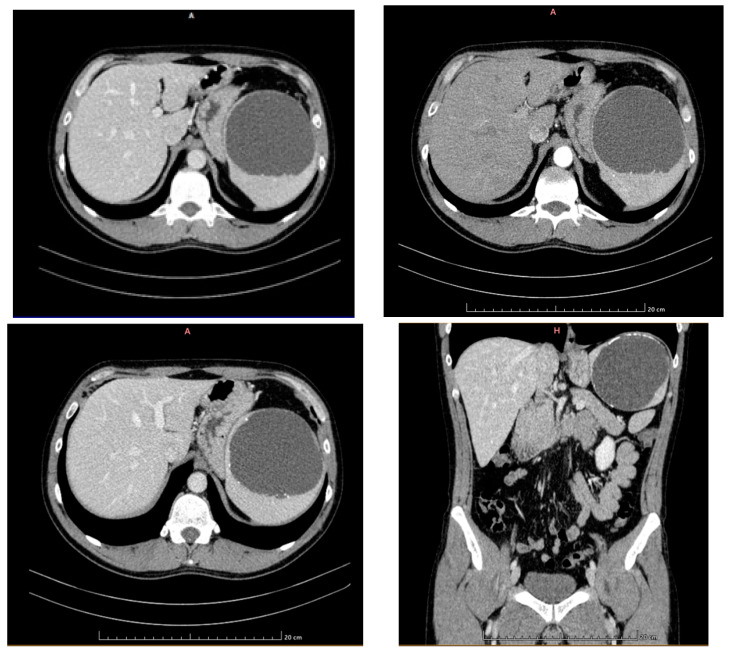
CT examination (axial /reconstruction in the coronal plane) native and postcontrast. The spleen with a voluminous lesion of 98/100/97 mm (AP/T/CC), with liquid densities, with calcifications on the contour and without postcontrast changes, occupying the upper 2/3 from the spleen. No adjacent vesicles are present. The lesion exhibits a discrete mass effect on the gastric fornix.

Subsequently, the CT scan (Figure 3) reveals a liver with normal dimensions, with a discrete hypodense, imprecisely delimited, slightly inhomogeneous area in the VI^th^ segment, 9 mm in diameter, and visible at all times of the examination, except in the late phase when it is hyperdense. The gallbladder has liquid content, apparently without hyperdense stones, and a wall with normal thickness. The intrahepatic and extrahepatic bile ducts and the portal vein have normal calibre. The pancreas is within normal limits.

A splenomegaly of 145/99/105 mm (anteroposterior/transversal/craniocaudal diameters) with a voluminous lesion of 98/100/97 mm (AP/T/CC) with liquid densities and calcifications on the contour occupies the upper two thirds of the spleen. No adjacent vesicles are present. The lesion exhibits a discrete mass effect on the gastric fornix (Figure 3).

The kidneys are of normal size and morphology, symmetrical secretion and excretion, within normal time limits, without hyperdense calculi or dilatation of the pyelocaliceal apparatus.

The adrenal glands are of normal dimensions, without expansive formations.

Under the given conditions of visualization of the digestive tract, no parietal thickenings and pathological contrast uptake at the intestinal level are detected. There is a dolichosigmoid. Several diverticula are present at the level of the sigmoid and the descending colon, without signs of complications. Mesenteric fat has normal appearance. There is an absence of abdominal, pelvic, or groin adenopathies. The abdominal aorta has calcified atheromatous plaques. There is an absence of peritoneal fluid.

The bladder is half full, with homogeneous liquid content, and a normal wall. The prostate has normal dimensions of 31/38/34 mm (AP/T/CC). The abdominal wall is without changes. Arthritic changes in the spine were included in the scanned volume, without CT elements, suggestive of secondary bone lesions.

No pleuro-pulmonary changes on the thoracic sequences were included in the abdominal exploration.

The blood panel reveals a slight increase in the number of red blood cells (6.26 × 10^6^/mm^3^), no eosinophilia, and IgG determined for echinococcus has values of 1553 mg/dL (reference range 700–1600 mg/dL).

After discussion with the patient and psychological support (young patient who is concerned about the possibility of losing an organ, i.e., spleen), a surgical approach is decided. We have also considered the living environment as possibly favourable for the appearance of the hydatid cyst and then adapted the surgical tactic.

**Figure 4:**
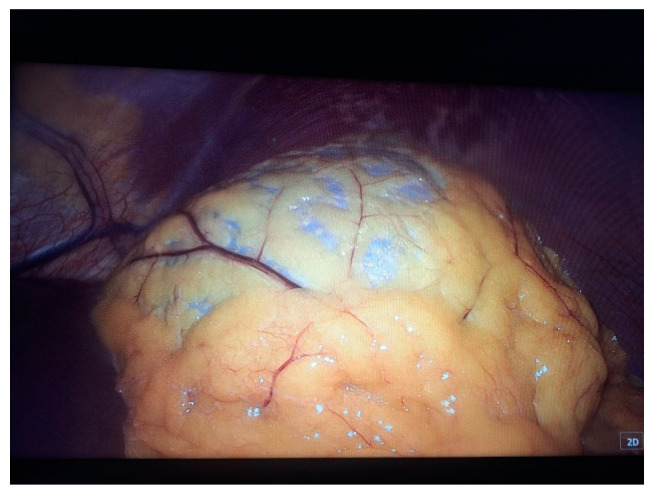
Laparoscopic intraoperative aspect. In the left hypochondrium, a white lesion, under tension, occupying the upper pole and the inner edge of the spleen was highlighted.

**Figure 5:**
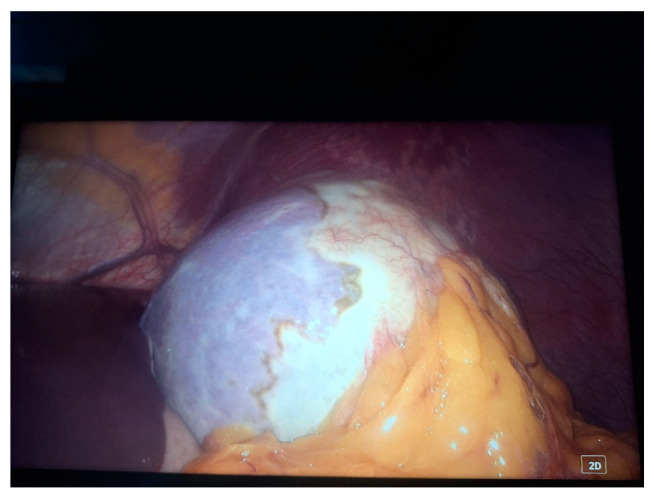
Laparoscopic intraoperative aspect after mobilization of the greater omentum.

**Figure 6:**
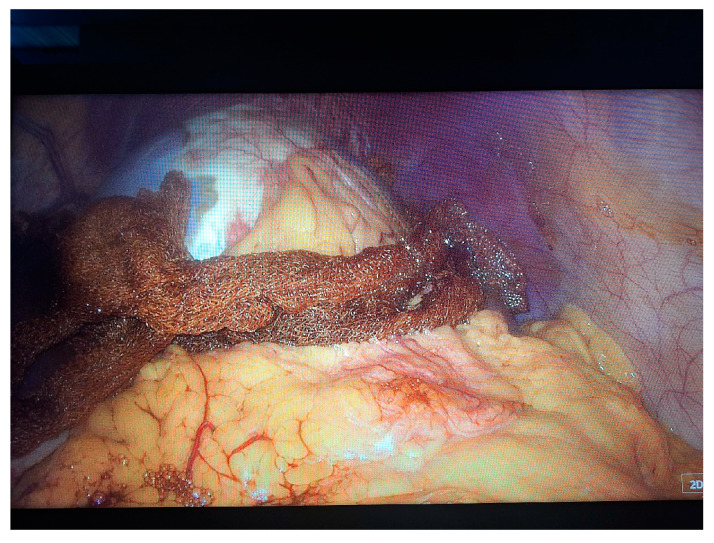
Laparoscopic intraoperative aspect. Isolation of the lesion with gauzes richly soaked with betadine^®^ 20%.

**Figure 7:**
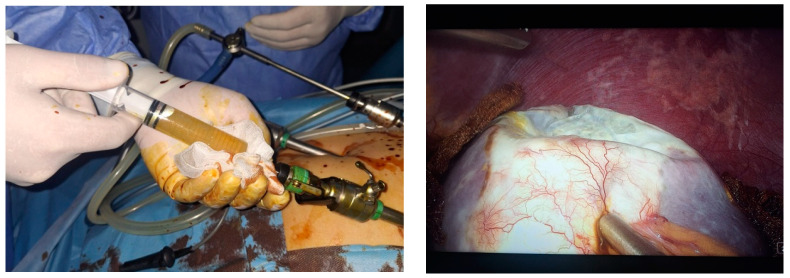
Laparoscopic intraoperative appearance, direct approach through aspiration puncture of the cavity with the highlighting of the liquid with a macroscopic serous appearance.

**Figure 8:**
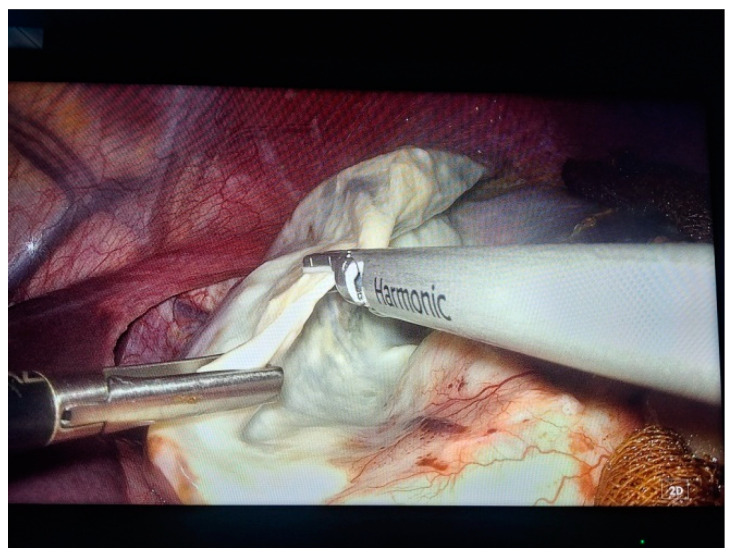
Laparoscopic intraoperative aspect. Fenestration of the cyst.

**Figure 9:**
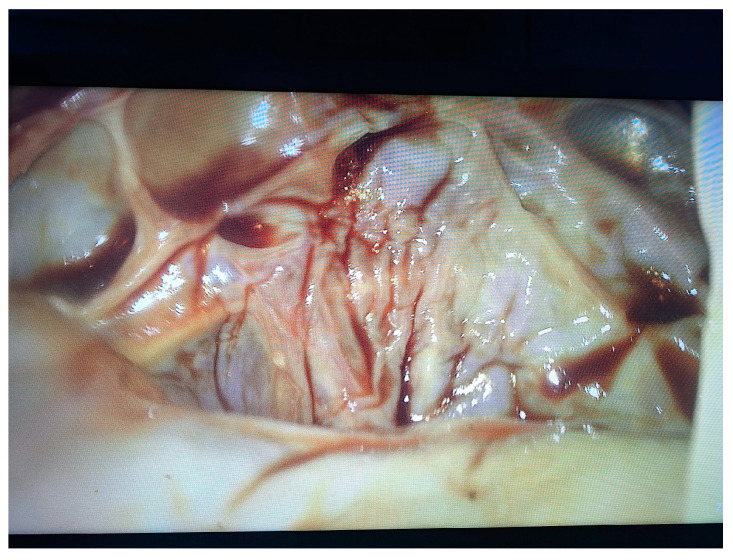
Laparoscopic intraoperative aspect. Inspection of the cavity, trabecular appearance of the wall.

**Figure 10:**
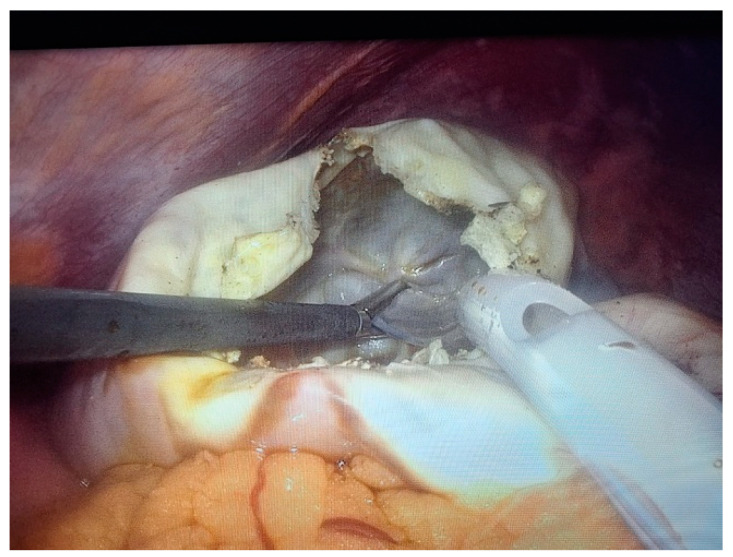
Laparoscopic intraoperative aspect. Cavity drainage with drainage tube.

**Figure 11:**
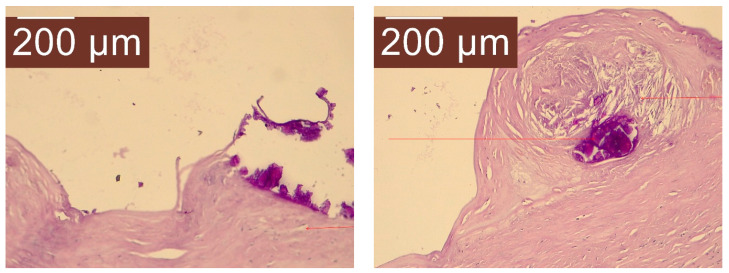
Microscopic appearance of the resection piece. Calcium deposits and acicular cholesterol crystals highlighted by arrows.

Two 10 mm trocars were placed supraumbilically and in the left hypochondrium and one 5 mm epigastric on the xiphoumbilical line. During laparoscopic exploration of the peritoneal cavity, in the left hypochondrium, a white lesion was identified, occupying the upper pole and the inner edge of the spleen (Figure 4 and Figure 5). The lesion was isolated with gauzes richly soaked with betadine^®^ (Figure 6). Since the free surface of the cyst was clearly visible, we did not initially dissect the hilum of the spleen as a preventive measure, but rather approached the cavity directly through puncture (Figure 7), the macroscopic appearance of the aspirated liquid (serous) not being able to differentiate between simple and uncomplicated hydatid cysts. After complete aspiration (Figure 8), the remaining cavity was washed with 20% betadine solution. Afterwards, the cyst was fenestrated with using the Harmonic scalpel ^®^(Ethicon Inc., Raritan, NJ, USA) (Figure 9), the inner cavity inspected (Figure 10) and subsequently drained with a soft silicone drainage tube (Figure 11).

The patient did not require hospitalization in intensive care, the drainage at 48 h allowed the suppression of the tube, and the discharge was decided on the third postoperative day.

The anatomopathological result (Figure 11) specifies at macroscopic examination: hard tissue fragment, pearly white, 2.5/1.5 cm.

Microscopic examination reveals that the examined fragment represented by the cyst wall formed by connective tissue, without identifying the covering epithelium. Numerous calcium deposits and acicular cholesterol crystals can be detected in the wall.

As such, the pathology findings are most likely associated with a primitive splenic cyst.

## 3. Discussion

The are numerous classifications of splenic cysts, such as primary or secondary; parasitic or non-parasitic and true or false cysts; non-neoplastic and neoplastic (2). Primary or true cysts are further sub-divided into parasitic cysts, commonly caused by Echinococcus granulosus, and nonparasitic cysts including epidermoid, dermoid, and endothelial cysts, whose aetiology is not fully understood [3,4]. In endemic areas, parasitic cysts, particularly those caused by Echinococcus granulosus, are the most common type of primary splenic cysts [3]. Nonparasitic cysts are largely attributed to congenital malformations or acquired conditions like trauma, infarction, inflammation, infections such as bacterial endocarditis, systemic lupus erythematosus, or malaria or neoplasms [3]. Secondary cysts, also known as pseudocysts (lacking the epithelial lining), typically result from previous spleen damage such as trauma or splenic infarction [5,6]. Neoplastic splenic cysts can be either benign or malignant, whereas non-neoplastic splenic cysts include post-traumatic (pseudocysts), congenital, inflammatory, and vascular cysts. Congenital cysts are true cysts lined by various types of epithelia. Various bacterial, fungal, or parasitic organisms can also determine splenic abscess formation.

However, most cases are idiopathic—meaning the exact cause is unknown. The risk factors for splenic cysts have not yet been thoroughly investigated due to the cysts’ rarity.

The clinical presentation of splenic cysts varies greatly. Many individuals are asymptomatic with cysts incidentally discovered during routine medical imaging studies, such as abdominal ultrasounds or CT scans [4]. Small splenic cysts are usually asymptomatic. Larger splenic cysts or those that cause stretching of the spleen’s capsule can lead to symptoms related to the mass effect on surrounding organs, such as a feeling of fullness or discomfort in the upper left abdomen left thorax, nausea, vomiting, and early satiety [7]. Some patients describe a dull, aching pain in the left upper quadrant, which may be intermittent. Larger cysts, however, may present with epigastric fullness, palpable mass, splenomegaly, abdominal pain or discomfort [7]. Other non-specific symptoms include flatulence, constipation, fatigue, pain in the left shoulder or left side of the neck, or can present more telltale symptoms as they enlarge. Occasionally, in more severe cases, splenic cysts may present with complications such as infections, anaphylactic shock, fistula, acute peritonitis, hemoperitoneum, and empyema secondary to the rupture of the cyst [2,4,8]. The physical exam may reveal an enlarged spleen. In cases of significant cyst enlargement, a palpable mass may be felt by a healthcare provider during a physical examination. The mass is typically located in the left upper quadrant of the abdomen. Pain is not a common symptom of splenic cysts, but it can occur if the cyst becomes infected, ruptures, or haemorrhages. An infected cyst may cause fever, chills, and severe abdominal pain.

Large splenic cysts may disrupt the normal architecture of the spleen, potentially affecting its hematologic functions. In some cases, splenic cysts have been associated with mild thrombocytopenia (low platelet count) or anaemia.

Although rare, splenic cysts can lead to serious complications such as cyst rupture, infection, or haemorrhage. Ruptured cysts can cause sudden, severe abdominal pain and may lead to peritonitis, requiring immediate medical attention. Depending on the underlying cause of the splenic cyst, patients may experience secondary symptoms related to the specific aetiology. For instance, in cases of parasitic cysts (e.g., echinococcosis), patients may exhibit allergic reactions or anaphylaxis.

Blood tests can aid in the diagnosis and evaluation of splenic cysts. Elevated white blood cell counts, especially in the presence of fever, may indicate infection within the cyst. Furthermore, certain serological tests can help diagnose parasitic cysts, such as *Echinococcus* spp., by detecting specific antibodies or antigens in the blood. Laboratory tests may reveal nonspecific elevated serum carbohydrate antigen (CA) 19–9 and carcinoembryonic antigen (CEA) levels [5]; serological tests and several imaging modalities such as US, CT, or magnetic resonance imaging are valuable in the differential diagnosis [2].

Imaging plays a significant role in the diagnosis of splenic cysts. Ultrasonography, computed tomography (CT), and magnetic resonance imaging (MRI) are frequently relied upon [9]. These imaging tests allow doctors to examine the spleen thoroughly and detect cysts, their location, and size.

Ultrasound is often the first technique employed due to its accessibility and non-invasiveness [10]. It is a non-invasive, cost-effective, and readily available tool. It can accurately evaluate cyst size, position, and whether the cyst is simple (filled with fluid) or complex (with internal structures, septations, or solid components) [11]. On ultrasound, splenic cysts appear as well-defined, anechoic (fluid-filled) structures within the spleen. Doppler imaging can assess blood flow in the cyst and the surrounding tissue. CT scan can provide critical information, such as cyst size, location, and wall thickness, that aids in differentiating cyst types [12]. It can identify calcifications and potential complications such as bleeding within the cyst, rupture, or infection [13]. CT scans provide detailed cross-sectional images of the spleen and are highly effective in characterizing splenic cysts. CT scans can differentiate between cystic lesions and solid masses, and their relationship with nearby structures. MRI offers a more precise differentiation between types of cysts by analysing the cyst’s content. It offers excellent soft tissue contrast and can distinguish between different cystic lesions within the spleen. Additionally, MRI can assess vascular involvement and rule out other abdominal pathologies. MRI can also assess complications such as cyst rupture or infection [14].

To exclude malignancy, fine-needle aspiration (FNA) or biopsy can be performed [9]. However, due to the risk of cyst rupture and dissemination of potential malign cells, or parasites if the possibility of a parasitic cyst is raised, the procedure is used sparingly [15].

Endoscopic ultrasound combines endoscopy and ultrasound to provide high-resolution images of the spleen and surrounding structures. It can help visualize the cyst’s location, size, and characteristics, making it useful for diagnostic and therapeutic purposes, such as cyst drainage or injection of sclerosing agents [16].

Molecular analysis examining the presence of epithelial lining cells can aid in discerning between primary and secondary cysts [17]. Additionally, serologic tests can help diagnose echinococcosis in cases of suspected parasitic cysts. In uncertain cases, diagnostic laparoscopy might be necessary [18].

The differential diagnosis of splenic cysts includes a variety of other conditions, such as solid tumours of the spleen, such as haemangioma, lymphangioma, or lymphoma, infectious cysts, such as hydatid cyst or pyogenic abscess, pancreatic pseudocyst, metastatic disease to the spleen, splenic infarction.

Management strategies for splenic cysts are highly influenced by the type and size of the cyst, clinical presentation, and associated complications [1].

Historically, total splenectomy has been the preferred treatment method for larger, multiple, deep-located splenic cysts. However, given the immunologic role of the spleen and increased risk and severity of post splenectomy infections, spleen-preserving minimally invasive procedures are currently recommended. These procedures include partial splenectomy (laparoscopic or open), total cystectomy, percutaneous sclerotherapy, marsupialization, and fenestration, and are recommended in the case of superficially located cysts, especially in children and young adults [2,19]. Asymptomatic, small cysts may be managed conservatively with regular monitoring. Interventional procedures such as percutaneous drainage, sclerosis, or fenestration may be considered given the specific circumstances, while surgical intervention (partial or complete splenectomy) is reserved for large cysts, symptomatic cases, or when malignancy cannot be excluded [1,20].

Recently, laparoscopic techniques have been increasingly applied to manage splenic cysts [21]. Splenic cysts are infrequent and typically benign entities often discovered incidentally. Their effective management has undergone a substantial revamp in recent years with the advent of the laparoscopic approach [22]. Historically, splenectomy was employed primarily as a treatment modality for splenic cysts. Nonetheless, concerns regarding the potential for overwhelming post-splenectomy infection in patients, especially in the young, provoked the search for spleen-preserving therapeutic modalities [23]. The advent of laparoscopic technology into surgery has triggered a paradigm shift in the management of splenic cysts towards a minimally invasive, spleen-preserving approach. The surgical technique involves unroofing the cyst and removing the cyst wall followed by argon beam coagulation of the remaining cystic lining [24]. A study by Uchiyama et al. (2003) [25] demonstrated that laparoscopic fenestration or partial cystectomy allows for cystic decompression, preserves spleen function, and reduces morbidity compared to splenectomy. Similarly, a study published in the British Journal of Surgery underpins the safety and efficacy of the laparoscopic approach even in the management of giant splenic cysts [26]. Another study by Han et al. (2019) indicated that laparoscopic partial splenectomy appears to be a safe and feasible approach for managing benign, non-parasitic cysts. The team reported outstanding results with minimal surgical complications and short recovery times [27].

In the management of splenic cysts, the laparoscopic approach has emerged as a valuable option because of its potential benefits over traditional open surgery [24]. This minimally invasive surgical method offers several advantages, including lower postoperative pain, a shorter hospitalization, quicker recovery time, and improved cosmetic results [24]. Laparoscopic intervention for splenic cysts is typically indicated when the cysts are larger than 5 cm, persistently symptomatic, or associated with complications such as rupture [28]. Cysts showing a suspicion of malignancy also justify expedited laparoscopic intervention to obtain histopathology [28]. Partial splenectomy and cyst fenestration are the two most employed laparoscopic techniques [29]. In partial splenectomy, the affected portion of the spleen is removed while leaving the healthy tissue intact. This procedure aims to preserve splenic function and limit the risk of overwhelming post-splenectomy infection (OPSI) [29]. Cyst fenestration or pericystectomy is indicated for cysts located superficially or involving less than 50% of the spleen. It entails opening the cyst and removing the inner lining, thereby preventing a recurrence [29].

Laparoscopic intervention has shown favourable outcomes in the management of splenic cysts. A systematic review by Touloumis et al. reported overall success rates reaching up to 96% for laparoscopic fenestration and 100% for laparoscopic partial splenectomy [30]. Furthermore, studies have suggested low recurrence rates (7–33%) and minimal postoperative complications [30].

Despite the advantages, laparoscopic intervention is not devoid of challenges and contraindications. Factors such as adhesion, poor patient condition, and large cyst size can increase the complexity of the procedure [4]. Moreover, patients with bleeding disorders, severe cardiorespiratory conditions, or previous abdominal surgery might be deemed unfit for laparoscopic intervention [31,32]. Although the evidence supports the effectiveness and safety of the laparoscopic approach, operator experience and patient selection remain paramount to ensure favourable outcomes. Future research efforts should focus on enhancing the scope, safety, and success rates of the laparoscopic management of splenic cysts.

Recent years have witnessed considerable advancements in the treatment of splenic cysts. Developments in endoscopic techniques, laparoscopic procedures, and minimally invasive approaches have expanded treatment options [1]. Furthermore, promising results have been observed with novel therapies including percutaneous ablation techniques [1]. Despite advancements, managing recurrent cysts remains a challenge. New approaches such as the installation of sclerosing agents are on trial, while the role of laparoscopic marsupialization continues to be evaluated [33,34,35].

## 4. Conclusions

Despite being rare entities, splenic cysts pose significant diagnostic challenges. Timely diagnosis and suitable management are integral for optimizing patient outcomes. The field of splenic cyst management has been revolutionized with advancements in imaging and minimally invasive interventions, but further research and clinical trials are needed to develop evidence-based guidelines for optimal management. Our case report presents a primitive splenic cyst that was classically benign, solitary, homogeneous, and anechoic, which was well suited for a conservative laparoscopic approach. However, if malignancy is a consideration, complete cyst excision with splenectomy or cystectomy with frozen section confirmation should be considered. Also, in the case of a large unilocular splenic cyst, a diagnosis of epidermoid cyst of the spleen should also be considered. Most non-parasitic splenic cysts have specific features on CT scanning to differentiate them from parasitic cysts. Laparoscopic partial excision with marsupialisation, as presented, represents an appropriate therapeutic approach, and method of treatment, which can result in the safe resolution of the condition, a shorter hospital stay, and improved patient satisfaction.

## Data Availability

The data presented in this study are available on request from the corresponding author.
